# Ultrafast and Deep Saliva Proteome Reveals the Dynamic of Human Saliva With Aging by Orbitrap Astral Mass Spectrometer

**DOI:** 10.1049/nbt2/6616433

**Published:** 2025-07-07

**Authors:** Lingling Jiao, Huilin Dong, Changjian Wu, Jing Liu, Chenhui Wang, Yi Cao, Fan Cao, Ying Zhu, Huaiyuan Zhu

**Affiliations:** ^1^China Tobacco Jiangsu Industrial Co. Ltd., Nanjing, China; ^2^School of Chemistry and Chemical Engineering, Southeast University, Nanjing, China

**Keywords:** age, mass spectrometry, proteomic analysis, saliva

## Abstract

Saliva has already proven to be a prospective diagnostic bioresource for both early disease detection and timely intervention due to its easy accessibility, noninvasiveness, and reproducibility. However, the in-depth identification of salivary proteins needs to be further improved. Until now, only 3427 proteins are included in the human salivary proteome (HSP), which is far from the millions of proteins that make up humans. Here, we set out to quantitatively map the HSP in rapid and in-depth Orbitrap Astral mass spectrometer (MS) and coronal nanomagnetic bead–based proteomics workflow. Our study reported 5937 salivary proteins, which was about 73% more than that recorded in HSP. Moreover, we compared the differences between the young and aged salivary proteins. The predominant functions of the upregulated proteins in the young were related to motor proteins and cardiomyopathy, whereas those of the aged were primarily upregulated with oxidation reaction, as well as neurodegenerative disorders. It is the first study to carry out salivary proteomics using a fast and deep Orbitrap Astral MS and remarkably enlarged the number of proteins with HSP, furthermore, salivary proteomics was found to be characterized in the young and aged. With the rapidly advancing MS and proteomics technologies, we believe that salivary protein biomarkers will be more promising for clinical diagnosis and prognosis of human diseases in the future.

## 1. Introduction

Traditionally, the substrate sources used for the analysis of diagnostic disease materials have been mostly serum or plasma, while they provide timely information on the current circulating concentration of the analyte [1]. Saliva analysis has attracted increasing attention in recent years, particularly from clinicians and researchers, who consider saliva as a noninvasive, easily accessible, and stress-free alternative biofluid to blood [2]. Whole saliva was a mixed biofluid that was derived predominantly from three pairs of major salivary glands (parotid, submandibular, and sublingual glands), with the remainder from thousands and millions of minor salivary glands located at various oral mucosal sites [3]. Saliva, including biochemical indicators of microbiota, RNA, DNA, and proteins, was regarded as a major component of the oral host defense system and constitutes the first line of defense against physical, chemical, and biological stimuli [4]. Our previous study found that whole saliva and saliva without cells presented different proteomic profiles, with whole saliva containing more protein species [5].

As a “mirror” of the body, saliva can be used for noninvasive detection and evaluation of point-of-care diagnostic potential, including oral and systemic diseases [6, 7]. Over the past two decades, saliva has been a more attractive diagnostic substance for its rich biocomponents, including hormones, immunoglobulins, and metabolites [4, 8]. Recently, in addition to determining oral health and disease, saliva tests have also been demonstrated with examples from the fields of endocrinology, infectious diseases, drug monitoring, metabolic and immunity diseases, neurological diseases, and cancers [1, 6, 9]. Due to the COVID-19 epidemic, saliva testing has proven to be a rapid, accurate, reliable, and reproducible test [10, 11]. In addition, saliva diagnostics may also be used to detect human immunodeficiency virus (HIV), and human papillomavirus (HPV), as well as for assessing hormones for daily routine [8, 12, 13].

Each day, humans produce approximately 0.5–1.5 L of saliva, constituting abundant biological omic components, especially thousands of proteins [14]. Nevertheless, there was a strong association between functions and constituents of saliva, and numerous salivary proteins engaged in more than one function, including digestion, mastication and deglutition, protection (antifungal, bacterial, and viral activity), defense (spiting and oxidative stress), water balance, and drug testing [15, 16]. Current research has contributed to understanding the salivary proteome, but the identification of novel and potentially diagnostic biomarkers remains limited. Meanwhile, the exact numbers of the salivary proteome remain unknown, with the human salivary proteome (HSP; https://salivaryproteome.org) estimated to suggest more than 3000 proteins.

Studies have found a rich exchange of metabolic substances between the salivary and blood circulatory systems. Moreover, many of the proteins detected in blood were also momentarily detected in saliva, these illustrated that saliva could be considered as a proxy for measuring diseases [17, 18]. However, there is still a challenge of salivary from laboratory research to clinical diagnostics, which is limited by a lack of the width and depth of detectable biomarkers in saliva. Advances in the use of saliva as a diagnostic biofluid have been facilitated by current technological developments in saliva omics, especially proteomic quantification methods.

Improving proteomic performance can be achieved in sample preparation to increase the detectable abundance of proteins and adopt advanced acquisition modes. For the sample preparation, we use untargeted proteome profiling nanoparticle-based affinity binders (nanobinders) for enriching low-abundant proteins in saliva. More recently, a novel type of mass spectrometry has been described—the Orbitrap Asymmetric Track Lossless analyzer (Astral), which can achieve high resolving powers (~80,000) and mass accuracy (5 ppm), and MS/MS scan speeds up to 200 Hz [19]. Here we describe the use of Proteonano assay and Orbitrap Astral MS instrument for the analysis of saliva proteome between the young and the aged. Our approach, which combined data-independent acquisition [20] proved to be time-efficient and achieved coverage of 5937 protein groups within a 14-min DIA run, while there were 3983 proteins not recorded in HSP. These marked a significant jump from current state-of-the-art salivary proteomic methodologies.

## 2. Materials and Methods

### 2.1. Saliva Collection

The study was ethically approved by the Ethics Committee of Zhongda Hospital, Southeast University, China. And we obtained informed consent from all participants. To minimize variability from systemic health conditions, exclusion criteria included: shift workers current medication; body mass index (BMI) greater than 30 kg/m^2^; personal history of cancer, diabetes, autoimmune disease, dental caries, oral disease or ulcers, and other medical conditions that might influence salivary protein measurements. Inclusion criteria were healthy adults (18–60 years old) with no self-reported chronic diseases or acute infections at the time of sampling. All male participants were recruited from the healthy population at Zhongda Hospital with 35 young (18–34 years old) and 42 aged (35–60 years old) males (Table 1). Participants were instructed not to eat, drink, smoke, or chew gum for 1 h before saliva collection. Before collection, the mouth was rinsed with water to remove oral tissue and debris. From 9:00 to 9:30 a.m., a saliva sample of approximately 1 mL was collected in an unstimulated state by spitting into a 50 mL vial placed on ice [21, 22]. Immediately, the saliva was added with PMSF (ST507, Beyotime, CN) and phosphatase inhibitor cocktail (04906837001, Roche, CH) to prevent protein from degrading. The samples were then stored at −80 °C until further use.

### 2.2. Protein Corona Preparation and Proteomic Analysis

The Nanoparticle kit (PN001-6, Proteonano Saliva Proteome Kit, Nanomics Biotech, CN) was sonicated for 5 min (40 kHz), then nanoparticles and mixed well. To form the protein corona [20], 100 μL of nanoparticle suspension was mixed with 25 μL of saliva samples in microtiter plates. The plates were sealed and incubated at 37°C for 1 h with shaking at 800 rpm. After incubation, the plate was placed on top of a magnetic collection device for 5 min to draw down the nanoparticle. The supernatant containing the nanocorona, unbound proteins was aspirated by pipetting. The protein corona was washed three times with 200 μL of wash buffer. To digest the proteins bound onto nanoparticles, a trypsin digestion kit (V511A, Promega, USA) was used according to protocols provided by the vendor. In brief, the digestion was performed by adding dithiothreitol, iodoacetamide, and trypsin, and the plates were incubated at 37°C for 16 h with shaking at 500 rpm. After stopping the digestion process with the addition of the supplied stop buffer, the nanoparticles were removed from the reaction by magnetic collection. After the digested peptides were desalted, the samples were then vacuum-dried and resuspended in 50 μL of 0.1% formic acid (695076, Thermo Fisher Scientific, USA). The concentration of peptides was measured by Nanodrop one from Thermo Fisher Scientific and was then ready for MS analysis.

### 2.3. Data Independent Acquisition Mass Spectrometry Analysis

Samples were analyzed on an Orbitrap Astral (Thermo Fisher Scientific, USA) in DIA mode. For each sample, 200 ng of peptides were analyzed on the Orbitrap Astral coupled with a Vanquish Neo UHPLC ultrahigh performance liquid chromatography system (Thermo Fisher Scientific, USA). Liquid chromatograph buffers were prepared as follows: Buffer A (0.1% formic acid in Milli-Q water (*v*/*v*)) and Buffer B (80% acetonitrile and 0.1% formic acid in Milli-Q water (*v*/*v*)). A 200 ng aliquots of each sample were loaded onto a trap column (300 μm × 5 mm, PepMap Neo Trap Cartridge, 174500, Thermo Fisher Scientific, USA). The peptides were eluted from the EASY-Spray HPLC column (2 µm, 0.150 mm × 150 mm, ES906, Thermo Fisher Scientific, USA) at a constant flow rate of 1.8 µL/min with a linear gradient from 4% Buffer B to 4% Buffer B in 0.3 min, from 4% Buffer B to 8% Buffer B in 0.7 min, from 8% Buffer B to 22.5% Buffer B in 6.7 min, from 22.5% Buffer B to 35% Buffer B in 3.7 min, from 35% Buffer B to 55% Buffer B in 0.4 min, from 55% Buffer B to 99% Buffer B in 0.5 min, and finally to 99% Buffer B within 0.7 min. The column was always kept at a constant temperature of 55°C.

The data were acquired using an easy spray source operated in positive mode with a spray voltage of 1.9 kV, a capillary temperature at 290°C, and a funnel radio frequency at 40°C. The MS was operated in DIA mode. A scan cycle comprised a full MS scan (*m*/*z* range from 380 to 980, with a maximum ion injection time of 5 ms, a resolution of 240,000, and an automatic gain control (AGC) value of 500%). MS survey scan was followed by MS/MS DIA scan events using the following parameters: Astral detector MS2 Activation Type is HCD, isolation window is 2 m/z, normalized collision energy is 25 eV, the scanning range of parent ion is 150–2000 *m*/*z*, RF lens is 40%, AGC target is 500%, the maximum IT value is 3 ms, and the loop control time is 0.6 s.

### 2.4. Database Search

The original mass spectrometry data were RAW files, the Spectronaut Pulsar 19.0 (Biognosys, CH) utilized default factory settings for database identification and quantitative analysis. Normalization strategy defined as local normalization and quantity MS-level set to use MS2. Related parameters and descriptions are shown in Table 2.

### 2.5. Statistical Analyses

A total of 5937 expressed proteins were identified from collected saliva (Table S1). Student's *t*-test was used to compare the two groups, and Benjamini–Hochberg correction was applied to control the proportion of false positives across all comparisons. Differential expression analysis utilized log_2_ fold change (|Log FC| > 0.5) and a significance threshold of *p* < 0.05 to determine differentially expressed proteins (DEPs). Annotation of all identified proteins was conducted by R studio (version 4.3.3) through the ggplots package with GO (http://www.blast2go.com/b2ghome; http://geneontology.org/) and KEGG pathway (http://www.genome.jp/kegg/). Proteins were subjected to GO and KEGG enrichment analyses.

## 3. Results

### 3.1. Identification of Salivary Proteome

**Figure 1:**
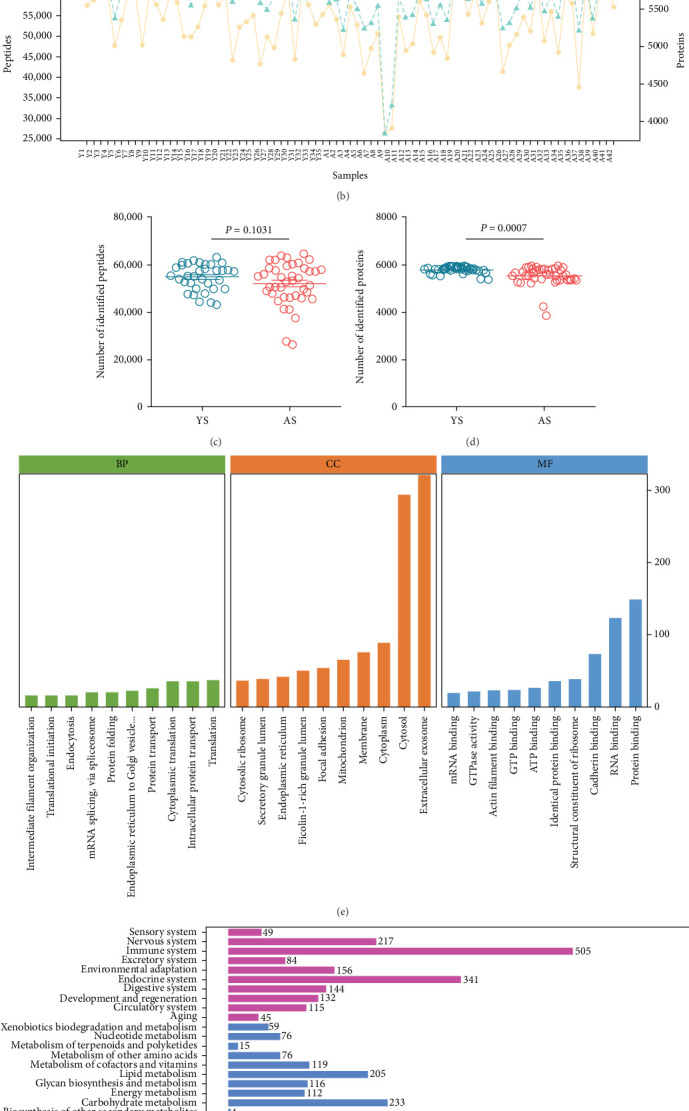
Detection and identification of the salivary proteome for 77 volunteers from 35 young and 42 aged males. (A) Workflow for salivary proteomes. (B) Quantification of peptide and protein frequencies in each sample. Quantification of peptides (C) and proteins (D) in the young (YS) and aged (AS) groups, respectively. Data are means ± SEM. *t*-tests were performed. Biological functions (E) and KEGG pathways (F) of the proteins in saliva.

As an ideal source of body fluid in a noninvasive manner, saliva was rich in different biological components including many proteins, nuclear acids, metabolites, and some cellular debris. To analyze saliva proteomics, saliva samples were processed on the following workflow in Figure 1A. First, all salivary samples were collected from the healthy participants grouped as the young and aged groups. The fresh saliva was then enriched and separated by nanoparticles to obtain the concentrated peptides ready for MS analysis. As shown in Figure 1B, 5937 proteins were discovered successfully by Orbitrap Astral MS. Although there was a significant difference in statistics, 5903 salivary proteins were identified in the aged groups, just 186 proteins more than in the young. While no significant difference in the expression number of peptide fingerprint spectra between young and aged groups (Figure 1C,D). The salivary proteins we identified here were mainly involved in signal transduction, infective disease, and the nervous system, especially neurodegeneration, and they may also contribute to the exosome, cytosol, and protein binding (Figure 1E,F). To the best of our knowledge, this would be the highest number of human salivary proteins reported up to now and showed that saliva has many functions that remain to be explored and validated.

### 3.2. Characteristics of Salivary Proteome

**Figure 2:**
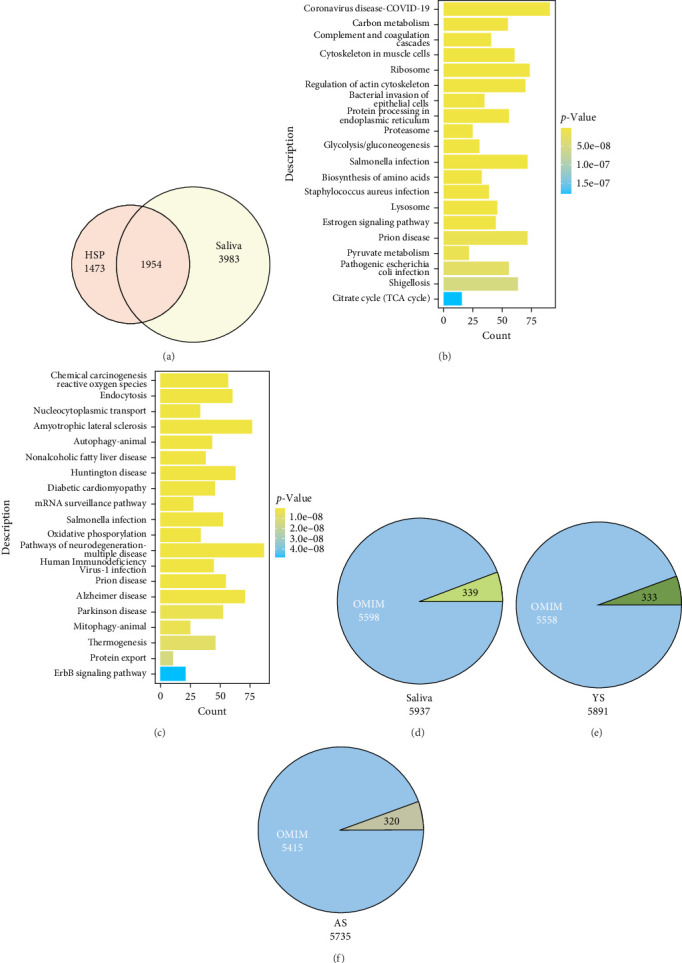
Characteristics of the identified proteins in human saliva. (A) Venn diagram illustrating the overlap of identified proteins referring to the HSP database. (B) KEGG pathways of the identified salivary proteins included in the HSP. (C) KEGG pathways of the identified salivary proteins excluded in the HSP. (D–F): Comparison of the identified proteins registered in the OMIM database. The blue area shows the number of the corresponding genes registered in the OMIM database among the identified proteins in saliva (D), YS (E), and AS (F) groups.

There were 1954 overlaps with the HSP database, while 3983 identified proteins by Orbitrap Astral MS were not recorded in the HSP (Figure 2A). For the overlap parts, most proteins were related to coronavirus disease, carbon metabolism, and prion disease (Figure 2B). For the unique species in our detection, the proteins were involved cell-related pathways, such as endocytosis, autophagy, and mitophagy, and neurodegenerative diseases, such as amyotrophic lateral sclerosis (ALS), prion disease, Alzheimer's disease (AD), Parkinson's disease (PD), and Huntington's disease (HD) [23] (Figure 2C). Moreover, to evaluate the potential applications of our salivary proteome analysis in the diagnosis of genetically related diseases, the detected salivary proteins were furtherly referred to Online Mendelian Inheritance in Man (OMIM; https://www.omim.org) database (Figure 2D–F), which contains causative genes of human genetic disorders and will give us more relationship between phenotype and gene-coded proteins [24]. Among the detected proteins, 5598 proteins in this study have been registered in the OMIM, and these illustrated that much higher numbers of the genetic-disorder proteins in salivary. For the samples from the different age groups, the registered proteins in the OMIM were 5558 for the young and 5415 for the aged, respectively. Distinct disease associations characterize gene sets across OMIM, AS, and YS databases. Beyond the overlapping genes between OMIM and AS, YS-specific genes exhibit significant enrichment for neuromuscular and metabolic pathologies: (1) MYOT- and CASQ1-associated myopathies [25, 26]; (2) CSRP3-mediated cardiomyopathies [27]; (3) LCAT-related norum disease, a rare autosomal recessive lipid metabolism disorder [28]. Conversely, AS-specific genes annotated in OMIM demonstrate predominant connections to neurological and mitochondrial mechanisms: (1) peripheral neuropathy-associated SLC25A46 mutations [29]; (2) NPRL3 variants linked to familial focal epilepsy [30]; (3) mitochondrial regulatory defects involving MT-ND2/6, SDHC, and TIMM22 [31, 32]. These results suggested that the salivary proteome we reported here not only expanded the known proteins in human saliva, but more importantly, dug out the therapeutic potential of saliva for diagnosing and monitoring diseases.

### 3.3. Characteristics of the DEPs Between the Young and Aged Group

**Figure 3:**
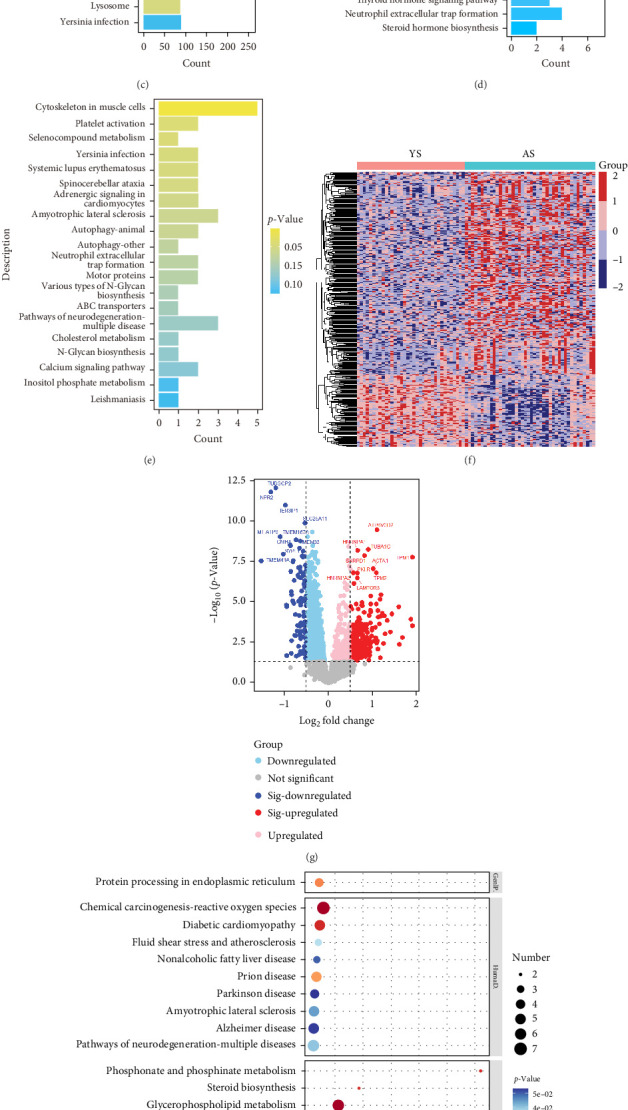
Characteristics of the differential proteins between the young and aged. (A) Heatmap with correlation coefficients among four groups. The same number indicated the same healthy volunteers. (B) Venn diagram illustrating the overlap of identified proteins between the YS and AS. (C) KEGG pathway in the YS and AS coexpressed proteins. (D) KEGG pathway of proteins that were only expressed in YS, but not in AS. (E) KEGG pathway of proteins that were only expressed in AS, but not in YS. (F) Heatmap of differentially expressed proteins between YS and AS. (G) Volcano plot of differentially expressed proteins between YS and AS. (H, I) Top 20 KEGG pathways of proteins that were upregulated (H) or downregulated (I) in AS compared with YS.

These above-rich data were obtained by Orbitrap Astral MS with an ultrafast and high throughput, which implies a wealth of information about the dynamic expression of HSP depending on the ages and other factors. The expression distribution difference and the correlation analysis of salivary proteins from different age groups were deeply investigated to gain their respective dynamics. The quantitative performance of the salivary samples collected from the young and aged groups was shown in Figure 3A, revealing a positive correlation with good coefficients for all samples. The overlap proteins were up to 5689 in the young and the aged groups with the possible function involved in neurodegenerative disease and cell death-related pathways (Figure 3B,C). The unique proteins in the young were related to glycosaminoglycan biosynthesis and biosynthesis of cofactors, while those unique to the aged were prone to the cytoskeleton in muscle cells (Figure 3D,E).

Comparative analysis of salivary proteomes between young and aged cohorts identified 360 DEPs, with 265 proteins downregulated and 95 upregulated in aged individuals. Prominently downregulated proteins (TUBGCP2, NPR2, MT-ATP6, SLC25A11, and so on) were enriched in cytoskeletal dynamics and cardiomyopathy pathways, while upregulated proteins (ATP6V0D2, HNRNPA1, TPM1, and so on) associated with oxidative stress responses and neurodegenerative disorders (AD and PD; Figure 3F–I). These age-dependent proteomic shifts highlight saliva's potential to reflect systemic aging processes and underscore the necessity of age-matched cohort design in salivary biomarker studies to ensure diagnostic accuracy.

## 4. Discussion

Here, we show that a nanomagnetic bead low abundance enrichment kit coupled with an Orbitrap Astral MS provided a platform for rapid and in-depth salivary proteome analysis (Figure 1A). The Proteonano™ Kit was composed of AI-designed polypeptides that could bind, and enrich low-abundance proteins in saliva with high specificity and sensitivity [20],. This capability combined with the Orbitrap Astral analyzer's high-quality accuracy, mass resolution, and sensitive ion detection allows for reliable localization of low abundance peptides as evidenced by the system's analytical performance for low overall peptide loadings. In summary, this approach collected the most in-depth HSP profile to date in only 25 μL fresh saliva and a 14 min run time of MS. This opens more possibilities for exploring salivary proteomic profiles.

High accessibility and noninvasive collection of saliva make it the attractive body fluid of biomarker research. Saliva-based testing has been considered a promising and noninvasive tool for on-site, remote, and real-time window into systemic health status. As for the well-known diagnostic methods for assessing salivary biomarker levels, notably enzyme-linked immunoassay (Elisa), and radioimmunoassay (RIA), these methods are usually time-consuming and low effective based on the specific binding of antigen and antibody [33, 34]. The untargeted detect proteins commonly used LC-MS/MS, which can only process one sample at a time for about 1 h or more [35, 36]. Therefore, the large-scale and deep analysis of human salivary biomarkers remains undestroyed and to fulfill the diagnostic value and clinical potential of saliva, saliva-specific analytical and diagnostic tools need to be developed and optimized [37]. Here we combined the Proteonano Saliva Proteome Kit, which was specially optimized for saliva, and the Orbitrap Astral MS platform to enhance salivary proteome and biomarker discovery. More surprisingly, 5937 proteins were detected, which was nearly double than 3427 proteins recorded in HSP (Figure 2A) [38]. And we also explore the difference between the young and the aged population.

It is the first time that nanoparticle-based affinity binders for low abundant protein enrichment and ultrahigh efficiency Orbitrap Astral MS have been made available for salivary proteomics. The approach of saliva proteome we conducted in this study, which combined DIA code [20], proved to be time-efficient and achieved coverage of more peptides and protein groups. Here we identified a total of 5937 proteins with only 25 μL of saliva and 14 min run of MS time, which was the smallest sample volume and the fastest analyzed time reported (200 µL [39] and 180 µL [40]), and our results not only overlap well with HSP, but more importantly, we identified 3983 proteins that were not currently included in this database. The function of these extended proteins was oxidation reaction and cell death-related pathways (endocytosis, autophagy, and mitophagy) and neurodegenerative diseases, including ALS, prion disease, AD, PD, and HD, these indicated that saliva has many functions that defy excavation and verification (Figure 2C). With these data, we present, to our knowledge, the deepest and most human saliva proteome reported in a single cohort. Together with the high accuracy of protein structures predicted by alpha fold [23], we believe that the physiological functions and clinical diagnosis of saliva will be further revolutionized.

Saliva shows circadian rhythmic changes across the 24-h cycle in humans, including its secretion and components, especially the hormone [22]. Given the importance of hormones in regulating normal circadian rhythms, salivary secretion during aging may also reveal age-related biomarkers. Studies have reported that saliva from aged subjects showed significant changes. A study showed that the concentration of mucins, both MG1 and MG2, decreased with aging [41]. Others reported that there were no significant correlations between mucin levels and age [42, 43], but the advanced oxidative protein products were found to increase with aging [44]. In our cohort, while the aged group showed only 186 additional salivary proteins compared to the young group, differential expression analysis revealed 360 age-dependent proteins (Figure 3G). Downregulated proteins in the aged group (TUBGCP2 and SLC25A11) were enriched in cytoskeletal dynamics and cardiomyopathy pathways, whereas upregulated proteins (ATP6V0D2 and TPM1) associated with oxidative stress and neurodegenerative disorders (Figure 3H,I). Strikingly, aged-unique proteins were linked to neurodegenerative pathways (Figure 3E), aligning with evidence that aging is the primary risk factor for neurodegeneration and that salivary biomarkers may be potential to reflect neurological dysfunction [9, 45, 46].

Though saliva testing shows its unique diagnostic value in many disorders, these breakthroughs mostly depend on the good correlation of the biomarker in blood. One study found that people with shared saliva had more intimacy with each other [47]. Saliva is becoming more and more important to the systemic health. These results have gone beyond what we have known about saliva. We believe in the possibility of salivary gland organoid cultures, which will accelerate further knowledge of saliva [48, 49]. With the significant advancements that have been noted in the development of salivary detection and diagnostic tools, the mechanisms of systemic diseases reflected in saliva are also becoming more explicit. Salivary biomarkers for the detection and diagnostics of systemic diseases would have insight into care, health disparities, and global health.

These salivary aging signatures both parallel and diverge from blood proteome trends. Like blood studies, saliva showed upregulated oxidative stress and neurodegeneration markers—hallmarks of systemic “inflammaging” [50–52]. However, while blood aging signatures often emphasize immune dysregulation (e.g., elevated pro-inflammatory cytokines) and coagulation cascades, saliva-specific changes highlighted cytoskeletal modeling (e.g., TUBGCP2 and TPM1) and mitochondrial function (MT-ATP6), potentially reflecting tissue-specific aging in salivary glands or oral cavity homeostasis. Conversely, the downregulation of cardiomyopathy-related proteins (NPR2 and SLC25A11) aligns with blood-based findings of declining cardiac markers in aging, suggesting conserved cross-tissue aging mechanisms. This positions saliva as a complementary biofluid for aging research, capturing both systemic and niche-specific pathways. Future studies integrating paired saliva–blood samples and longitudinal designs could disentangle localized vs. systemic aging drivers, advancing saliva's diagnostic utility.

Sexual dimorphism in the salivary proteome may originate from autosomal sex-biased gene (SBG) expression patterns, as emerging evidence demonstrates that approximately 90% of SBGs reside on autosomes and display population-specific regulatory dynamics influencing salivary protein networks [53]. The omission of female participants in the current study design specifically addresses confounding hormonal variables—notably menstrual cycle phase, pregnancy status, and exogenous hormone use (e.g., contraceptives)—which robustly modulate immune-related proteins and enzymatic activity in saliva [54]. Future investigations should integrate sex-stratified analyses to resolve these differences, given the proteomic divergence observed in female-specific physiological contexts (e.g., gestation) and experimental models with controlled hormonal milieus.

## Figures and Tables

**Table 1:** The characteristics of salivary cohort between the young and aged.

Gender	Male	
Group	Young saliva (YS)	Aged saliva (AS)
Age, years (mean ± SD)	28.91 ± 3.958	41.67 ± 7.021
BMI, kg/m^2^ (mean ± SD)	25.26 ± 2.658	25.40 ± 2.784
Participants, *N*	35	42

**Table 2:** Quantitative parameters for protein identification.

Item	Value
Enzyme	Trypsin/P
Max missed cleavages	2
MS1 accuracy	±10 ppm
Mass accuracy	0.02 Da
Fixed modifications	Carbamidomethyl
Variable modifications	Oxidation
Database	Homo_sapiens.GRCh38.pep.all.fasta
Peptide FDR	≤0.01

## Data Availability

The data that support the findings of this study are available from the corresponding author upon reasonable request.

## Supporting Information

Supporting Information Table S1: Identification of salivary proteome in the young and the aged.
